# Possible Therapeutics Effects of Ayahuasca, a Psychedelic Compound

**DOI:** 10.1192/j.eurpsy.2022.2260

**Published:** 2022-09-01

**Authors:** L. Silva, L. Bravo

**Affiliations:** Unidade Local de Saúde do Baixo Alentejo, Psychiatry, Beja, Portugal

**Keywords:** Ayahuasca, psychedelics, DMT, drug addiction

## Abstract

**Introduction:**

Ayahuasca is an hallucinogenic tea prepared from the Amazonian vine *Banisteriopsis caapi* and the leaves of the plant *Psychotria Viridis.* Banisteriopsis caapi contains monoamine oxidase inhibitors (MAO) that render the N, N-dimethyltryptamine (DMT) of Psychotria Viridis active. This brew is being used as a sacrament in syncretic religions in urban areas of Brazil and worldwide with the purpose of enabling a spiritual experience as well as healing for a variety of conditions such as drug addiction, depression and anxiety disorders.

**Objectives:**

This work aims to provide an understanding on the potential benefits of ayahuasca in psychiatric symptoms, as well as its neuropsychological functioning, neuroimaging and adverse events.

**Methods:**

A non-systematic review was performed on PubMed database and Google Scholar, using the key words “Ayahuasca, Drug Addiction, Psychedelics, DMT, Neuroplasticity”. The review included experimental studies in humans, observational studies, systemic review articles and clinical trials.

**Results:**

In a randomized placebo-controlled trial, ayahuasca had a significant antidepressant effect when compared to the placebo group. Long term ayahuasca usage was associated with structural alterations in the medial parts of the brain with no evidence of increased psychopathology or worse neuropsychological functioning. According to reviewed studies the incident of prolonged psychotic reaction among ayahuasca users is rare and the causal relation with ayahuasca is sometimes difficult to establish.

**Conclusions:**

Despite the promising results, more controlled double-blinded studies with larger populations are necessary to better understand the therapeutic potentials and side effects of ayahuasca.

**Disclosure:**

No significant relationships.

